# Effects of nutritional guidance on frailty in older adults: A systematic review

**DOI:** 10.1016/j.jnha.2025.100756

**Published:** 2025-12-11

**Authors:** Tatsuya Koyama, Junko Nohara, Mieko Nakamura

**Affiliations:** a National Institutes of Biomedical Innovation, Health and Nutrition, National Institute of Health and Nutrition, Japan; b Kio University, Japan

**Keywords:** Frailty, Nutritional guidance, Dietary education, Older adults, Systematic review

## Abstract

**Background:**

Frailty is a prevalent geriatric syndrome associated with risk of disability, hospitalization, and mortality. Despite nutrition and exercise playing central roles in maintaining muscle mass and function, the specific effects of nutritional guidance, distinct from supplementation, remain unclear.

**Objective:**

This systematic review aimed to evaluate the effectiveness of nutritional guidance interventions on frailty in community-dwelling older adults.

**Methods:**

A systematic search in MEDLINE (PubMed) identified relevant randomized controlled trials (RCTs) published up to April 2025. Eligible studies included those with participants aged ≥65 years who received nutritional guidance, defined as dietary counseling or education without supplementation, with frailty status as the primary outcome measure. The risk of bias was assessed using Cochrane RoB 2.0.

**Results:**

From 211 initial records, 11 relevant RCTs were included in the analyses. The interventions varied in duration (12 weeks to 8 years), delivery (individual or group sessions), and implementers (dietitians, health professionals, or non-professionals). Short-term interventions produced mixed results, whereas long-term programs, particularly those combined with exercise, showed more consistent improvements in frailty measures. Individually tailored and professionally delivered interventions were generally more effective. However, substantial heterogeneity in intervention design, frailty definitions, and outcome measures limited comparability across studies. Few trials quantitatively assessed dietary intake, restricting mechanistic understanding.

**Conclusion:**

Nutritional guidance can help prevent or improve frailty in older adults, especially when implemented as a long-term, individualized, and professionally delivered program, ideally combined with physical activity. Future research should adopt standardized frailty criteria, reliable dietary assessment methods, and multidisciplinary approaches to strengthen the evidence and inform sustainable strategies for healthy aging.

## 1 Introduction

Aging is a global health concern that affects all individuals. According to the World Health Organization, the number of people aged 60 years and older is projected to increase from 1 billion in 2019 to 1.4 billion by 2030, and more than 2.1 billion by 2050 [1]. Aging is associated with various physical, cognitive, and social changes affecting morbidity and mortality and accompanied by a progressive decline in muscle mass, strength, and physical performance. From approximately 50 years of age, individuals are estimated to lose 1–2% of leg muscle mass and 1.5–5% of leg strength per year [2]. These physiological changes contribute to an increased vulnerability to adverse health outcomes.

Frailty is a clinical syndrome characterized by a decline in physiological reserves across multiple organ systems. While sarcopenia—manifested as slowness, weakness, exhaustion, low activity, and weight loss—is an important feature, frailty extends beyond muscle loss to include cognitive, psychological, and social domains [3]. Frail individuals are at increased risk of adverse outcomes, including falls, fractures, disability, hospitalization, and mortality [4]. The prevalence of frailty among older adults varies widely, ranging from 4% to 59%, depending on the criteria and population studied [5]. Given its significant impact on health and quality of life, frailty prevention has become a major priority in geriatric care and public health.

Various interventions including physical activity, pharmacological treatment, and nutritional strategies have been explored to prevent or improve frailty in older adults. Adequate nutrient intake plays a central role in preserving muscle mass and function [6]. Several systematic reviews have investigated the effects of nutritional supplementation, such as protein, vitamin D, and amino acids, on frailty prevention and improvement [[7], [8], [9]]. However, nutritional guidance (i.e., dietary counseling or education aimed at improving dietary habits) may also influence frailty status by promoting long-term behavioral changes and healthier eating patterns.

Despite growing interest in lifestyle-based interventions, there is a lack of comprehensive research on the effectiveness of nutritional guidance on frailty among community-dwelling older adults. To date, no systematic review has specifically focused on the impact of nutritional guidance, independent of supplementation, on frailty prevention and improvement. Therefore, this systematic review aimed to evaluate and summarize the current evidence regarding the effectiveness of nutritional guidance in improving frailty status in older community-dwelling individuals.

## 2 Methods

This systematic review was conducted in accordance with the Preferred Reporting Items for Systematic Reviews and Meta-Analyses guidelines [10]. This review was registered with PROSPERO (International Prospective Register of Systematic Reviews) (registration number: CRD420251035502).

### 2.1 Search strategy

We included all primary source randomized controlled trials (RCTs) published in English or Japanese up till April 2025 that met the following eligibility criteria:

• Population: community-dwelling older adults, generally defined as mean age ≥65 years. Therefore, studies enrolling participants aged ≥60 years were also included if they otherwise met the eligibility criteria, in recognition of variations in age thresholds across trials.
• Intervention: nutritional guidance, defined as nutritional advice or counseling, excluding nutritional supplementation, or diet delivery as the sole intervention of interest
• Outcome: frailty status

The MEDLINE electronic database (PubMed) was used as the primary source to identify eligible studies. Although we also trialed searches in other bibliographic databases, no additional eligible peer-reviewed journal articles were identified. We did not extend our search to non-peer-reviewed reports or unpublished studies (grey literature). The search was conducted in April 2025. The following keywords were used: (aged OR elderly OR senior) AND (“nutrition* advice” OR “nutrition* modification” OR “nutrition* intervention” OR “nutrition* counseling” OR “nutrition* guidance” OR “nutrition* education” OR “diet* therapy” OR “diet* advice” OR “diet* education” OR “diet* modification” OR “diet* counseling” OR “diet* guidance” OR “lifestyle intervention” OR “lifestyle modification” OR “feeding behavior” OR diet OR eating OR “eating together” OR “shared meal*” OR “self-monitoring” OR “house calls”) AND (frailty OR frail* OR “frailty syndrome” OR “functionally impaired” OR “frail elderly”) AND (“randomized controlled trials as topic” OR “randomized controlled trial”).

### 2.2 Eligibility criteria for the studies

Studies that fulfilled the following criteria were included in this review: (1) RCT design; (2) participants’ age ≥ 65 years; (3) participants living in the community; (4) nutritional guidance as an intervention in the experimental group; (5) outcomes measured frailty status; and (6) published in English or Japanese. The exclusion criteria were as follows: (1) study protocols, systematic reviews, or meta-analysis as study types; (2) participants living in hospitals or long-term care facilities; and (3) nutritional interventions that included dietary prescriptions, dietary oral supplements, or delivery of specific diets only.

### 2.3 Study selection and data extraction

Two independent researchers conducted a preliminary screening of the identified literature by reviewing the abstracts and titles. Subsequently, the two researchers assessed and selected the full texts of potentially relevant articles based on the eligibility criteria. Any disagreements were resolved through discussions with the third researcher to reach a consensus.

The extracted data included study details (authors, year of publication, and sample size), participant characteristics (age and frailty criteria), intervention protocols (implementation of nutritional guidance, type of nutritional guidance, session length, session frequency, intervention duration, control group intervention), and relevant outcomes.

### 2.4 Assessment of risk of bias

Version 2 of the Cochrane risk-of-bias tool for randomized trials was used to assess the risk of bias [11]. This tool covers five domains of bias: bias arising from the randomization process, bias due to deviations from the intended interventions, bias due to missing outcome data, bias in the measurement of the outcome, and bias in the selection of reported results. The risk of bias was classified as low (with some concerns) or high for each item.

### 2.5 Data synthesis

We evaluated the feasibility of a meta-analysis but determined it was not appropriate due to substantial heterogeneity in frailty definitions and outcome measures (e.g., Fried’s phenotype, Clinical Frailty Scale, SHARE-FI, Check-List 15), clinical diversity of interventions (nutrition alone vs. multidomain; duration from 12 weeks to 8 years; professional vs. non-professional implementers), inconsistent follow-up times, and incomplete reporting of essential summary statistics (means with SDs, event counts, hazard ratios with SEs). In line with the Cochrane Handbook for Systematic Reviews of Interventions [12], we therefore conducted a structured narrative synthesis [13].

## 3 Results

### 3.1 Study selection

**Fig. 1:**
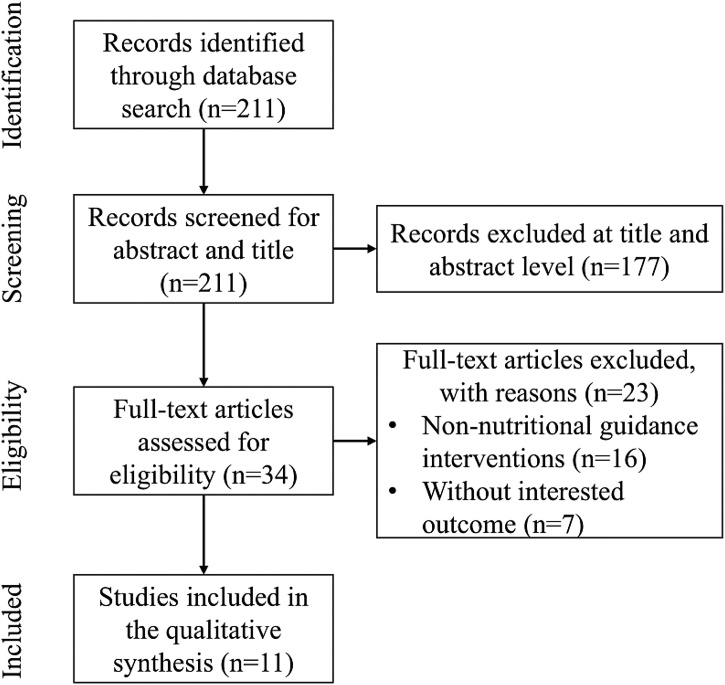
Flow diagram of the study selection process.

A flow diagram of the study selection process is presented in Fig. 1. The literature search identified 211 articles. After screening the titles and abstracts, 34 full-text articles were assessed for eligibility; 11 studies met the inclusion criteria and were included in this review.

### 3.2 Risk of bias in the included studies

The risk of bias assessment is summarized in Table 1. Most studies demonstrated a low risk of bias for deviations from intended interventions, missing outcome data, outcome measurements, and selection of reported results. However, some studies [16,18,20,22] were judged to have “some concerns” regarding bias arising from the randomization process because the details of the randomization methods were inadequately reported.

**Table 1:** Cochran Risk-of-Bias 2.0 assessment of the included studies.

	Risk of bias domains					
First author	D1	D2	D3	D4	D5	Overall
Ji [14]	Low	Low	Low	Low	Low	Low
Saarela [15]	Low	Low	Low	Low	Low	Low
Liang [16]	Low	Some concerns	Low	Low	Low	Some concerns
Wu [17]	Low	Low	Low	Low	Low	Low
Lee [18]	Low	Low	Low	Low	Low	Some concerns
Teh [19]	Low	Low	Low	Low	Low	Low
Huguet [20]	Low	Some concerns	Low	Low	Some concerns	Some concerns
Hsieh [21]	Low	Low	Low	Low	Low	Low
Barreto [22]	Low	Some concerns	Low	Low	Low	Some concerns
Seino [23]	Low	Low	Low	Low	Low	Low
Luger [24]	Low	Low	Low	Low	Low	Low

Domains: D1: Bias arising from the randomization process D2: Bias due to deviations from intended intervention D3: Bias due to missing outcome data D4: Bias in measurement of the outcome D5: Bias in selection of the reported result.

### 3.3 Characteristics of the included studies

Table 2 summarizes the characteristics of the 11 included studies, which were conducted in Korea [14], Finland [15], Taiwan [[16], [17], [18],^21^], New Zealand [19], Spain [20], France [22], Japan [23], and Austria [22]. Seven studies included older adults regardless of frailty [[14], [15], [16], [17], [18],^22^], none included only prefrail older adults, one study included frail older adults [19], and three studies included both prefrail and frail older adults [21,23,24].

**Table 2:** Summary of the study characteristics of the included studies.

First author, location	Sample characteristics: n, mean age ± SD, sex (female ratio)	Inclusion criteria	Exclusion criteria
Ji [14] Korea	IG: n = 21, 77.9 ± 4.5, 11 (52.4%) CG: n = 21, 78.2 ± 4.5, 11 (52.4%)	Aged ≥ 65 years, ambulatory with or without an assistive device, and living at home	Residing in nursing homes or hospitals or receiving nursing home-level care at home
Saarela [15], Finland	IG: n = 631, 69.5 ± 4.7, 286 (45.3%) CG: n = 629, 69.2 ± 4.7, 302 (48.0%)	Aged 60–77 years and have cardiovascular risk factors, aging and incidence of dementia risk score ≥ 6 points	Not reported
Liang [16], Taiwan	IG: n = 529, 75.3 ± 6.4, 397 (75.0%) CG: n = 525, 74.9 ± 6.4, 326 (62.1%)	Aged ≥ 65 years, subjective memory impairment and/or loss of one instrumental activity of daily living, and/or a timed 6-meter walk speed ≤ 1 m/sec	A history of dementia, severe hearing or visual impairment, documented major depression or anxiety, a major illness with a life expectancy of < 6 months, or other conditions affecting compliance Participants having total or partial dependence for activities of daily living or those institutionalized
Wu [17], Taiwan	IG: n = 87, 75.2 ± 0.7, 63 (72.4%) CG: n = 124, 73.1 ± 0.6, 98 (79. 0%)	Not reported	Communication disability, severe disease, dietary control at doctors’ instructions, or inability to walk 14 m independently
Lee [18], Taiwan	Robust group: IG: n = 57, 70.5 ± 4.4, 39 (68.4%); CG: n = 36, 70.3 ± 4.6, 15 (41.7%) Cognitive impairment, no dementia group: IG: n = 20, 70.7 ± 4.4, 13 (65.0%); CG: n = 30, 70.9 ± 5.0, 19 (63.3%) Mobility impairment, no disability group: IG: n = 50, 72.8 ± 5.6, 31 (62.0%); CG: n = 51, 72.5 ± 5.46, 30 (58.8%) Physio-cognitive decline syndrome group: IG: n = 48, 75.3 ± 6.6, 26 (54.2%); CG: n = 48, 73.4 + 6.3, 26 (54.2%)	Community-living people aged ≥ 65 years, with ≥ 3 chronic medical conditions	Terminal illness and/or severe disability, inability to communicate adequately with study staff, having malignancy and undergoing active chemotherapy, having life expectancy < 12 months, and currently institutionalized
Teh [19], New Zealand	Senior chef program group: n = 117, 80.0 ± 5.2, 63 (53.8%) Steady as you go program group: n = 118, 79.9 ± 4.9, 77 (65.2%) Combined group: n = 118, 79.8 ± 5.2, 70 (59%) Social group: n = 115, 81.4 ± 5.2, 68 (59.1%)	Pre-frail; aged ≥ 75 years (≥ 60 years for Māori and Pacific Peoples), without any terminal illness or advanced dementia, able to stand, medically safe to participate in low-intensity exercise, and able to use kitchen utensils safely	Not reported
Huguet [20], Spain	IG: n = 68, 88.8 ± 3.2, 47 (69.1%) CG: n = 67, 88.3 ± 3.4, 40 (59.7%)	Non-institutionalized patients, both men and women aged ≥ 80 years, pre-frailty	Prior diagnosis of advanced dementia, palliative care with a life expectancy < 6 months, clinically unstable, patients included in home care program, wheelchair users, severe sensory deficits
Hsieh [21], Taiwan	CG: n = 80, 72.5 ± 5.5, 29 (36.3%) Exercise group: n = 79, 72.0 ± 6.0, 33 (41.8%) Nutrition group: n = 83, 70.4 ± 5.3, 38 (45.8%) Combination group: n = 77, 71.6 ± 6.0, 27 (35.1%)	Aged ≥ 65 years; frail or pre-frail	Non-frail, unable to walk a 14-m distance independently, severe illnesses, severe depression, cognitive impairment, communication impairment, hospitalized or living in a nursing home, taking nutritional supplements
Barreto [22], France	Multidomain group: n = 816, 75.3 ± 4.3, 525 (64.3%) CG: n = 821, 75.3 ± 4.5, 534 (65.0%)	Community dwellers ≥ 70 years of age who met ≥ 1 of the following criteria: spontaneous memory complaints, limitations executing ≥ 1 instrumental activity of daily living, or gait speed < 0.8 m/s	Not reported
Seino [23], Japan	Immediate IG: n = 38, 74.9 ± 5.3, 14 (36.8%) Delayed IG: n = 39, 74.3 ± 5.6, 10 (25.6%)	Score of ≥ 2 on the Check-List 15	Routine participation in health promotion activities and presence of a serious or unstable illness
Luger [24], Austria	Physical training and nutritional IG: n = 39, 83.0 ± 8.1, 33 (84.6%) Social support group: n = 41, 82.5 ± 8.0, 34 (82.9%)	At risk of malnutrition or malnourished, prefrail or frail, ≥ 65 years, and able to walk	Impaired cognitive function, planned admission to a nursing home, undergoing chemo- or radiotherapy, comorbidities, chronic obstructive pulmonary disease stage III or IV, chronic kidney insufficiency, and persons classified as nursing level 6 or 7

SD, standard deviation; IG, intervention group; CG, control group.

### 3.4 Summary of findings from the included studies

Table 3 summarizes the nutritional guidance and results of the included studies. Regarding the criteria used to identify frailty, seven studies used the Fried frailty criteria [[15], [16], [17], [18], [19], [20],^22^]. The Fried phenotype defines frailty based on five components—unintentional weight loss, exhaustion, weakness (grip strength), slowness (walking speed), and low physical activity—and classifies individuals as frail (≥3 components), pre-frail (1–2 components), or robust (0 components).

**Table 3:** Summary of the outcomes of the included studies.

First author	Evaluation of intervention	Frailty definition	Frailty	Results
Ji [14]	12 weeks	Clinal frailty scale	Mean frailty index change (95% CI) Intervention -0.06 (−0.08 to −0.03), Control 0.03 (0.00 to 0.05)	Compared to the control group, improvement in frailty index was observed in the intervention group.
Saarela [15]	2 years	Fried’s phenotype	Change in frailty status Intervention: No frailty to no frailty 71.2%, no frailty to pre-frail or frail 14.0%, no frailty to no information 15.0% Pre-frail or frail to no frailty 34.4%, pre-frailty or frail to pre-frail or frail 39.2%, pre-frail or frail to no information 25.5% Control: No frailty to no frailty 69.5%, no frailty to pre-frail or frail 14.4%, no frailty to no information 16.0% Pre-frail or frail to no frailty 31.3%, pre-frailty or frail to pre-frail or frail 49.9%, pre-frail or frail to no information 19.0%	There was no difference between the intervention and control groups in the incidence of frailty among all participants.
Liang [16]	6 and 12 months	Fried’s phenotype	Mean difference in frailty index change between intervention and control (95% CI) 6 months −0.128 (−0.251 to −0.005) 12 months −0.152 (−0.281 to −0.024)	In the intervention group, there was a notable improvement in frailty status for older participants at 12 months.
Wu [17]	3 and 6 months	Fried’s phenotype	Mean changes in frailty status ± SEM from baseline 3 Months: Nutrition group −0.01 ± 0.05, Control −0.16 ± 0.06 6 Months: Nutrition group −0.02 ± 0.05, Control −0.06 ± 0.07	Frailty status was significantly lower for the nutrition group compared to the control group at 3 months, but this phenomenon disappeared at 6 months.
Lee [18]	3, 6, 9, and 12 months	Fried’s phenotype	Mean changes in frailty scores (95% CI) CIND group (overall difference −0.3, 95% CI − 0.5 to −0.1) MIND group (overall difference −0.3, 95% CI − 0.4 to −0.1)	Integrated multidomain primary care services significantly reduced frailty scores among participants with CIND and MIND groups across all timepoints.
Teh [19]	6, 12, and 24 months	Fried’s phenotype	Mean changes in frailty scores (95% CI) At 6-month follow-up SC group −0.310 (−0.622 to 0.002) SAYGO group −0.404 (−0.686 to −0.123) Combined group −0.164 (−0.445 to 0.118) At 24-month follow-up SC group −0.203 (−0.534 to 0.129) SAYGO group −0.077 (−0.406 to 0.252) Combined group −0.084 (−0.405 to 0.237)	At 6 months post-intervention, frailty scores improved in the Senior Chef program and Steady As You Go program groups, remained stable in the combined group, and worsened in the control group. By 12 months, scores in all groups returned near baseline, except in the control group, where frailty continued to worsen. At 24 months, scores increased in all groups, but those in the combined group were more likely to transition to a robust state, unlike the single-program or control groups.
Huguet [20]	12 and 36 months	Fried’s phenotype	At 12 months Intervention: Robust 14.7%, pre-frail 79.4%, frail 5.9% Control: Robust 1.5%, Pre-frail 76.1%, frail 22.4% At 36 months Intervention: Robust 14.7%, pre-frail 63.2%, frail 22.1% Control: Robust 1.5%, pre-frail 65.7%, frail 32.8%	At 36 months, in the intervention group, there was a lower percentage of frail patients compared with the control group. There was also a significant difference in the number of robust patients at both 12 and 36 months.
Hsieh [21]	12, and 36 months	Modified Fried’s phenotype	Difference in difference change of frailty scores from baseline in each intervention group minus change from baseline in control group (95% CI) At 1 month Exercise −0.06 (−0.24 to 0.11), Nutrition 0.10 (−0.08 to 0.28), Combination −0.07 (−0.25 to 0.11) At 3 months Exercise 0.11 (−0.07 to 0.29), Nutrition −0.06 (−0.24 to 0.11), Combination −0.03 (−0.21 to 0.15) At 6 months Exercise −0.23 (−0.41 to −0.05), Nutrition −0.28 (−0.46, −0.11), Combination −0.34 (−0.52, −0.16)	The exercise, nutrition, and combination groups revealed statistically significant improvement in the frailty scores after the 6-month follow-up compared with the control group.
Barreto [22]	3 years	Fried’s frailty phenotype	Mean difference in frailty index change between intervention and control (95% CI) 6 months −0.001 (−0.01 to 0.00) 1 year 0.00 (−0.01 to 0.00) 2 years 0.00 (−0.01 to 0.00) 3 years −0.01 (−0.02 to 0.00) Hazard ratio of frailty incidence (frailty incidence ≥ 0.25) 0.72 (0.55 to 0.93) (Control group was the reference category.)	The between-group adjusted mean difference indicated that the multidomain group had a significantly lower frailty score at 3 years compared with controls. The Cox model showed that compared with controls, the multidomain group had decreased risk of developing frailty.
Seino [23]	3 months	Check-list 15	Intervention effect of initial 3-month period (IIG change − DIG change) CL-15 − 0.36 (−0.74 to −0.03) Prevalence of pre-frailty or frailty 4.3% (−17.2%–25.7%) Prevalence of frailty only −23.5% (−40.4% to −6.7%)	As compared with the delayed intervention group, the immediate intervention group showed small but statistically significant reductions in the Check-list 15 and the prevalence of frailty only, but not in prefrailty/frailty at 3 months.
Luger [24]	12 weeks	Frailty instrument for primary care of the survey of health, ageing and retirement in Europe	Mean difference in frailty index change or prevalence of frailty (95% CI) (Control group was the reference category.) SHARE-FI −0.30 (−0.75 to 0.15) Odds ratio of frailty 0.80 (0.33–1.99)	There was no significant difference between the physical training and nutritional intervention groups and the social support group.

CI, confidence interval; CIND, cognitive impairment, no dementia; MIND, mobility impairment, no disability; SAYGO, Steady as you go program; SC, senior chef program; SHARe-FI, frailty Instrument for Primary Care of the Survey of Health, Ageing and Retirement in Europe.

Other studies employed alternative frailty classifications. One study used the Clinical Frailty Scale (CFS) [14], a global clinical judgement tool ranging from 1 (very fit) to 9 (terminally ill), which incorporates comorbidity, mobility, energy, and functional independence into an overall frailty rating. One trial applied a modified Fried phenotype [21], which uses the same conceptual domains as Fried et al. but employs locally adapted cutoffs or measurement procedures. The Check-List 15 [23] assesses multidimensional risk across physical, nutritional, social, and psychological domains; higher scores indicate greater frailty risk. Finally, one study used the SHARE-FI instrument [24], which classifies frailty based on self-reported exhaustion, loss of appetite, weakness, slowness, and low physical activity, combined with grip strength to generate a frailty score and categorical classification (frail, pre-frail, non-frail).

The intervention duration ranged from 12 weeks to 8 years. Multicomponent interventions were particularly prominent, with nutrition often integrated into broader programs that also included components of physical activity [15,16,18,24], cognitive training [16,18,24], and social engagement [19,24].

The review of nutritional guidance identified 11 studies with varying objectives and methodologies (Table 4). Of these, six aimed to promote well-balanced diets [[15], [16], [17],^20^,^21^,^23^], whereas three targeted increased protein intakes [17,23,24]. Regarding the implementers, four interventions were conducted by dietitians or nutritionists [[14], [15], [16],^21^], whereas three were conducted by research staff whose professional qualifications were not explicitly specified [18,19,21]. Educational methods included individual counseling in six studies [14,15,20,21,24] and group-based instruction in seven [[15], [16], [17], [18], [19],^22^,^23^]. The duration of a single nutrition education session varied substantially, ranging from 15 min [16,18,22] to 3 h [19].

**Table 4:** Summary of nutritional guidance implemented in the included studies.

First author	Goals	Implementer	Educational methods	Materials	Frequency
Ji [14]	Practical strategies for sarcopenia management	A single dietitian from the public health center	Individual nutritional education sessions	Not reported	Not reported
Saarela [15]	To achieve an adequate intake with a balanced diet	A study nutritionist	• Individual sessions including tailoring of the participant’s daily diet • Group meetings providing more information and support for facilitating lifestyle changes, and discussions and practical exercises, such as tools to assess one’s own dietary behavior (e.g. tests to assess fat or fiber intake)	Not reported	Individual counseling (three meetings during the first year) and group sessions (6 times during the first year and 1–3 times during the second year)
Liang [16]	• To foster health lifestyle behaviors and motivate participants to practice on their own at home • General information on healthy nutrition and diet based on the Dietary Guidelines of Taiwan • Balanced dietary choices including macronutrients and micronutrients • Maintaining adequate protein intake	A trained dietitian	Clusters of 20–50 people were divided into smaller groups of 5–8 participants per session to promote effective delivery	Taiwan Health Promotion Administration dietary guidelines	Month 1: Four sessions (15 min/week) Month 2: Two sessions (15 min/fortnight) Months 3–12: Ten sessions (15 min/month)
Wu [17]	To promote the Taiwanese Daily Food Guide for older adults in everyday practice, supplemented with the Taiwanese eating approach food choice principle associated with geriatric syndromes Each participant was encouraged to consume the correct servings of the six food groups at their individualized energy levels; to select diverse nutrient-dense foods from each food group; to prioritize protein foods (soy and beans, aquatic foods, eggs, poultry, and meat, in that order); and to drink a cup of tea, coffee, or caffeine-free herbal tea every day	Not reported	• In the first 5 weeks of intervention, participants received intervention via group activities on (1) know my plate and food groups, (2) whole grains and roots, (3) drinking teas with dairy, and nuts and seeds, (4) novel ways to eat fruits and vegetables, and (5) healthy breakfast ideas • In week 6, individual reports were provided about their dietary intake and frailty status assessed at baseline to motivate them to comply with the intervention • During weeks 7 and 8, participants were asked to take 1-day photos of their diet for conducting a photo-elicitation focus group	Several tools, such as WAKE Taiwan website for estimating energy levels, food models, and the three-dimensional dining plate designed by the Taiwanese Association of Diabetes Educators, were employed in the teaching	A weekly 1 h nutrition activity
Lee [18]	Not reported	Trial investigators	A group-based, in-person, non-computer-assisted multidomain intervention	National dietary guidance for older people	15 min
Teh [19]	Not reported	A trained facilitator	The 3-h session comprising a morning tea (or light snack) prepared by the facilitator, nutrition education, cooking together (hands on cooking in pairs) using provided utensils and cooking facilities and then sharing the meal cooked that day The nutrition education covered practical information about sourcing food, menu planning, budgeting, and shopping tips, and written information was provided to the attendees to take home	Not reported	A weekly 3 h session run for 8 weeks
Huguet [20]	Not reported	A primary healthcare nurse, expert on the Mediterranean diet	Individual advice	Not reported	Not reported
Hsieh [21]	To help participants consume a nutritious diet with appropriate distribution of the six food groups and achieve the recommend dietary allowance level of nutrients	A licensed dietitian	Two sessions of individual nutrition education	A set of customized dishware	Not reported
Barreto [22]	Not reported	Qualified trainers	• Small groups (6–8 participants) • Nutritional advice based on dietary guidelines established by the French National Nutrition and Health Program for older adults. Eight key guidelines were proposed during the first two months, offering specific recommendations for a healthy diet	Not reported	• 15 min for nutritional advice • First two months (two sessions a week for the first month and one session a week the second month). After the second month, sessions were planned monthly
Seino [23]	Improving dietary variety and protein intake	Not reported	The nutritional program consisted of: (i) a general lecture on the functions of nutrients and foods and the importance of dietary variety; (ii) practical activities to improve understanding of adequate intake with each dish and meal; (iii) group activities to teach participants simple methods of consuming a variety of foods and to regard meals as fun	A checklist to assess dietary variety and the Japanese Food Guide Spinning Top	30 min once every 2 weeks
Luger [24]	To ensure adequate fluid, protein, and energy intake, preferably by regular foods and beverages, without the use of nutritional supplements	Nonprofessional volunteers called buddies	• Buddies discussed nutrition-related messages. In total, eight nutrition-related messages could be discussed, including for individual goal setting and tools to reinforce self-efficacy • Participants were provided with ideas on how to enrich food with proteins	• The booklet designed by nutritional scientists included three main nutritional aspects: fluid intake, animal and plant protein intake, and energy intake • Recipes of dishes that are protein and energy rich	Buddies visited twice a week

**Fig. 2:**
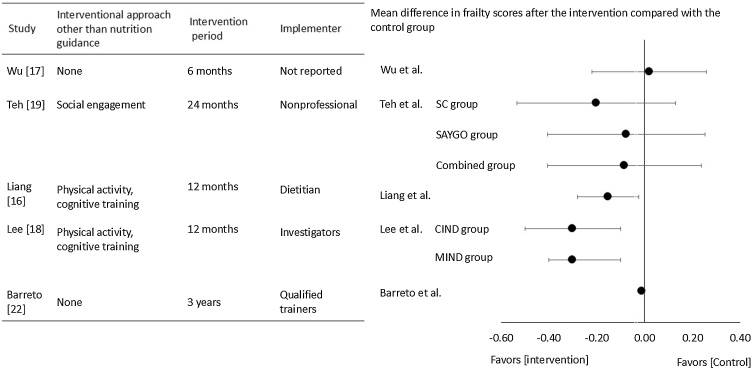
Summary of the five randomized controlled trials evaluating frailty using Fried’s phenotype. Regarding Studies by Saarela et al. and Hugest et al., since no Frailty scores were provided, it was omitted from the figure. Abbreviation: CIND, cognitive impairment, no dementia; MIND, mobility impairment, no disability; SAYGO, steady as you go program; SC, senior chef program.

When synthesized across studies, short-term interventions (<6 months) showed inconsistent results, with improvements in some trials [14,21] and null findings in others [17,19]. In contrast, long-term interventions (≥6 months) more consistently produced favorable changes in frailty outcomes [15,16,18,20,22], particularly when delivered by trained professionals [[14], [15], [16],[20], [21], [22]] and tailored to individual needs [14,15,21]. This pattern suggests that duration and professional expertise are key determinants of intervention effectiveness. Summary of studies of the five randomized controlled trials evaluating frailty using Fried’s phenotype is shown in Fig. 2.

## 4 Discussion

This review found few consistent associations between specific methods of nutritional guidance and frailty outcomes, likely reflecting intervention heterogeneity. Mechanistically, nutritional guidance may reduce frailty through promoting sustained dietary behavior change, particularly protein and micronutrient adequacy. Implementation factors—including professional delivery, cultural tailoring, and access to nutritious foods—are critical for real-world effectiveness. Clinically, these findings highlight the importance of integrating dietary counseling into multidisciplinary community programs, ideally combined with structured exercise.

Most studies included in this review combined multiple-strategy interventions. Consequently, it is difficult to disentangle the specific effects of nutritional counseling from co-interventions such as physical activity, cognitive training, and social engagement. This represents a key limitation that must be considered when interpreting the findings. As aging is associated with several physical, cognitive, and social changes, a multidisciplinary approach is recommended for older adults’ healthy living. Nutritional supplementation combined with resistance and/or endurance exercises has positive effects on muscle mass, muscle strength, and physical performance in frail and sarcopenic older adults [25] and in non-frail community-dwelling older adults [26]. The efficacy of interventions incorporating multiple components, such as exercise, has also been demonstrated in nutritional guidance.

This review found few consistent associations between the methods employed in nutritional guidance—whether delivered individually or in groups—and the duration of the sessions, with improvements in frailty. This inconsistency may reflect heterogeneity in the intervention content, differences in the baseline health and nutritional status of the participants, and variability in frailty case definitions and outcome measures.

One potential pathway by which nutritional guidance influences frailty is through behavioral changes in dietary intake; therefore, the precise measurement of dietary changes is crucial for understanding the mechanisms and evaluating interventions. However, relatively few trials included in our review incorporated quantitative dietary intake assessments as outcomes [17,23]. Conducting dietary assessments among older adults is generally considered challenging owing to cognitive decline, reduced comprehension, and the burden of self-reporting [27,28]. These factors pose significant barriers to accurate data collection. Consequently, there is a need to develop and standardize dietary assessment methods that are both low-burden and reliable for use in older populations for future research.

Taken together, these findings suggest that long-term, professionally delivered, and individually tailored nutritional guidance, especially when combined with structured physical activity, are more likely to yield clinically meaningful improvements in frailty-related outcomes in older adults. Nevertheless, the observed variability across trials indicates that effectiveness is also influenced by the specific content of the nutritional component (dietary advice and skills training), participant characteristics (baseline nutritional status, comorbidity, and cognitive function), adherence, and choice of outcome measures.

To enhance the efficacy of nutritional guidance, interventions should be carefully tailored to individual needs and preferences, and evidence-based dietary recommendations specific to frailty prevention and management should be incorporated. Practical components, including skills training in meal planning and food preparation, may strengthen the impact of such interventions. Furthermore, it is essential to address the potential barriers to dietary change such as limited access to nutritious foods and insufficient cooking abilities. Combining nutritional guidance with complementary strategies, such as structured physical activity programs, may provide synergistic benefits. Sustained support and follow-ups are critical for maintaining adherence and achieving long-term improvements. Future research is required to clarify the optimal components, duration, and modes of delivery of nutritional guidance interventions, with particular attention to the diverse needs of subgroups of older adults at risk of frailty.

The results of the included studies showed heterogeneous intervention effects. However, a consistent pattern was identified: long-term interventions (≥6 months), particularly those delivered by trained professionals and tailored to individual participants’ needs, tended to demonstrate more favorable effects on frailty outcomes. When interpreting this pattern, it is important to consider the risk of bias (RoB). Some trials reporting beneficial long-term effects were judged as having ‘some concerns’ regarding the randomization process, often due to insufficient reporting of allocation concealment, which may pose a risk of selection bias. Nevertheless, the beneficial pattern associated with long-term, professionally delivered, and individualized nutritional interventions was also observed in studies assessed as having a low overall risk of bias.

Therefore, while methodological rigor remains crucial, the consistency of findings across both low- and moderate-risk studies supports the interpretation that sustained, expert-led nutritional counseling may exert a genuine beneficial effect on frailty among community-dwelling older adults.

We carefully considered conducting a meta-analysis. However, marked heterogeneity in outcome definitions, intervention designs, and reporting formats, combined with missing data required for standardization, rendered a quantitative synthesis both statistically inappropriate and clinically uninterpretable. As advised in the *Cochrane Handbook* [12], pooling under such conditions risks generating misleading results. Instead, we followed established guidance for narrative synthesis [13], systematically identifying consistent patterns and considering study quality in our interpretation. This approach provides a more reliable representation of the current evidence base.

This systematic review has some limitations. First, our search was limited to peer-reviewed journal articles. Although exploratory searches in other databases were performed, they did not yield additional eligible articles beyond MEDLINE. We did not systematically review non-peer-reviewed reports or unpublished literature (grey literature), and thus relevant findings from these sources may have been overlooked, potentially introducing publication bias. Second, a critical issue in research involving older adults is the influence of individual lifestyle factors such as activities of daily living, dietary patterns, and physical activity behaviors. These lifestyle characteristics are often shaped by cultural and social contexts and can vary widely between countries. Therefore, the generalizability of the findings may be limited, and cultural implications should be considered when interpreting the results. Finally, substantial heterogeneity was observed among the included studies. Differences in the operational definitions of frailty or pre-frailty, types of nutritional guidance interventions, and outcome assessment methods limited direct comparisons between studies. This variability poses challenges in synthesizing the findings and drawing firm conclusions regarding the effectiveness of nutritional guidance on frailty in older adults.

## 5 Conclusion

Nutritional guidance holds promise in improving or mitigating frailty among older adults, particularly when integrated with structured exercises. However, current evidence is inconsistent because of methodological heterogeneity and contextual variability. Robust recommendations will require trials that adopt standardized frailty definitions and outcome sets, validated and low-burden dietary assessment methods, and multidisciplinary interventions that combine nutrition with exercise and practical support. Such efforts are necessary to develop effective, sustainable, and culturally adaptable programs that promote healthy aging and reduce the burden of frailty on individuals and healthcare systems.

## CRediT authorship contribution statement

Concept and design: TK, JN, MN. Acquisition, analysis, or interpretation of data: TK, JN. Drafting of the manuscript: TK. Critical revision of the manuscript for important intellectual content: JN, MN.

## Funding

None to disclose.

## Data availability

The datasets during and/or analyzed during the current study are available from the corresponding author upon reasonable request.

## Declaration of competing interest

All authors declare no conflict of interest.
